# Permeability of the extracellular space in the rat, mouse, and human brain

**DOI:** 10.21203/rs.3.rs-11368353/v1

**Published:** 2026-10-28

**Authors:** Aditya Ranjan, Yisen Guo, John H. Thomas, Douglas H. Kelley, Kimberly A. S. Boster

**Affiliations:** 1 Department of Mechanical Engineering, University of Rochester, Hopeman Engineering Building, Rochester, 14623, NY, USA.

**Keywords:** Extracellular space, Brain tissue, Permeability, Volume fraction, Kozeny-Carman

## Abstract

**Background:**

Brain tissue is a highly complex, heterogeneous, porous medium containing neurons, glial cells, blood vessels, and fluids. The interstitial spaces surrounding cells, referred to as the extracellular space (ECS), are critical transport pathways whose disruption is believed to play a role in neurological diseases. Therefore, understanding ECS fluid dynamics and the factors modulating flow there, such as permeability, is essential. Furthermore, because brain morphology, scale, and cellular density vary across species, understanding how permeability varies between species is critical for accurate physiological modeling.

**Methods:**

In our study, we investigated ECS permeability by performing computational fluid dynamics (CFD) simulations on three-dimensional electron microscopy data from rat, mouse, and human. We artificially varied the ECS volume fraction to investigate associated permeability variations, since volume fraction changes with arousal state. We also used a mesoscale computational model to simulate the waste clearance from the brain tissue.

**Results:**

The mean permeabilities of the ECS in rat, mouse, and human brain tissues, averaged across sampled locations, were approximately 18, 34, and 10 nm^**2**^, respectively. We compared our computational results with those predicted by the Kozeny-Carman equation, which consistently underestimated permeability. We developed an empirical relation to describe the variation of permeability with volume fraction within the range relevant to brain tissue.

**Conclusion:**

Our results show that the ECS permeability is of the same order of magnitude, approximately 10 nm^**2**^ across all species but varied several-fold across datasets. Hence, for models incorporating ECS permeability, a reasonable range of ECS permeability values for rats, mice, and humans is 10 nm^**2**^ – 100 nm^**2**^.

## Introduction

Brain waste clearance depends on the combined effects of diffusion and transport of cerebrospinal fluid (CSF). Approximately 80% of CSF is produced in the brain ventricles, with the remaining 20% arising from interstitial fluid transported across the blood-brain barrier (BBB) [1]. After flowing from the ventricles into the subarachnoid space, CSF enters the perivascular spaces and exchanges with the brain ECS through aquaporin-4 (AQP-4) water channels and gaps between astrocyte endfeet [2–4]. Within the ECS, this fluid is referred to as interstitial fluid (ISF). The movement of CSF and ISF, driven by mechanisms such as arterial wall pulsations [5, 6], slow vasomotion [7] and the sleep–wake cycle [8], facilitates the removal of metabolic waste products, including amyloid-*β* and tau proteins, from the brain [5]. Accordingly, the hydraulic permeability (or simply the permeability) of the ECS, which characterizes the ease of fluid movement through this space, is an important property for understanding transport processes within the brain.

Metabolic waste clearance is governed by advection, diffusion, and microglial degradation of waste [5, 9]. Early mouse studies attributed approximately 75% of extracellular amyloid-*β* clearance to the BBB and 10% to bulk ISF flow [10], whereas imaging studies, beginning with Iliff et al. [5], suggested a potentially substantial contribution from AQP4-dependent glymphatic flow. Determining the transport by advection and diffusion within the ECS remains challenging because current imaging techniques cannot measure fluid transport in deep regions of the brain with sufficient resolution. Consequently, fits to brain-wide clearance measurements [11], overall network models [12], and detailed computational models [13–16] have served as valuable tools for investigating fluid flow and solute transport within the brain. Recently, a physics-informed machine learning framework inferred brain-wide flow velocities, based on experimental data on tracer movement in the brain [17]. However, these modelling approaches rely on very uncertain estimates of the permeability of the ECS [18].

The ECS is not static: its volume fraction varies with arousal state [8], pathological conditions [19], and ageing [20]. Here, volume fraction (sometimes called porosity) is defined as the ratio of the volume of the ECS to the total tissue volume. While activity-dependent ion fluxes and osmotic gradients regulate localized cellular swelling and shrinkage, macroscale shifts in volume fraction are largely driven by noradrenergic signalling across sleep–wake cycles [8, 19]. These structural changes are expected to substantially influence permeability and fluid transport and, consequently, affect the efficiency of metabolic waste clearance. Therefore, accurately estimating the permeability under different arousal states is essential for understanding the mechanisms underlying brain waste clearance.

Characterizing how the ECS permeability differs across species is equally important. Such knowledge can improve our understanding of species-specific brain physiology and provide valuable insights for the design and optimization of brain-targeted drug delivery strategies.

Several previous studies have estimated the permeability of brain tissue. Basser [21] formulated a poroelastic model of fluid infusion into brain tissue, establishing a theoretical relation to estimate hydraulic permeability from steady-state pressure and flow rate. To illustrate the model’s predictions, the author adopted a gray matter hydraulic permeability value of 5 × 10^−9^ cm^4^ · dyn^−1^ · s^−1^ (or an intrinsic permeability of 4000 nm^2^ assuming the fluid dynamic viscosity to be 0.8 mPa · s) derived from previously published studies [22]. Morrison et al. [23] derived the gray matter hydraulic conductivity (1.65 × 10^−8^cm^4^ · dyn^−1^ · s^−1^) (or an intrinsic permeability of 13200 nm^2^) by matching their analytical model to the experimental results of Chen et al. [24]. Jin et al. [25] estimated the effective hydraulic conductance of the ECS by solving the Navier-Stokes equation in a two-dimensional Voronoi network model of the brain ECS. They reported a hydraulic conductance of ECS to be around 0.9 × 10^−9^cm^4^ · dyn^−1^ · s^−1^ (or an intrinsic permeability of 720 nm^2^). Holter et al. [26] performed simulations on the dataset of Kinney et al. [27] and found that the average permeability of rat brain tissue is 10.7 nm^2^. Experimental approaches for measuring the hydraulic permeability of brain tissue have also been explored; for example, Jamal et al. [28] performed ex vivo experiments on samples of ovine brain white matter by imposing a pressure drop across the tissue to drive fluid flow. They reported an average hydraulic permeability of approximately 70 nm^2^ perpendicular to the axons and 200 nm^2^ parallel to the axons, indicating substantial anisotropy in white matter permeability. Guo et al. [13] found that an ECS permeability of 3000 nm^2^ could explain the serum albumin clearance observed in the experiments of Cserr et al. [29]. Toscano et al. [17] used a machine learning model to estimate flow velocity and permeability from concentration distributions in mice. Their permeability estimates ranged from 100 nm^2^ to 1000 nm^2^.

Collectively, these studies reveal substantial differences in reported estimates of ECS permeability, highlighting the lack of consensus regarding its magnitude. This discrepancy underscores the need for a rigorous computational framework to determine ECS permeability directly from experimentally acquired electron microscopy datasets, across multiple species and brain regions.

In this study, we used publicly available segmentations of electron micrographs of rat [27], mouse [30], and human [31] brain tissue. The rat’s brain was fixed using transcardial perfusion, and the ECS was artificially inflated to achieve a volume fraction of 20%. The mouse and human datasets were acquired after immersion fixation. However, an osmotic agent was used during the preparation of the mouse dataset to preserve the ECS, resulting in an average ECS volume fraction of 15% [30]. The dataset for human brain tissue was prepared using immersion fixation without the use of an osmotic agent.

Previously, Holter et al. [26] estimated permeability through simulations based on electron micrographs of rat brain tissue. To our knowledge, however, permeability estimation from electron micrographs of mouse and human brain tissue has not yet been attempted.

Our simulations indicate that ECS permeability is comparable across the three species, with differences remaining within a few-fold rather than spanning an order of magnitude or more, across physiologically realistic ECS volume fractions. Our simulations also predict the extent to which ECS permeability varies across different arousal states. Furthermore, we compared values of the permeability determined with our simulations with values given by the standard Kozeny-Carman equation and found that this equation consistently predicts significantly smaller values of the permeability. We therefore propose a simple empirical formula that better captures the relationship between permeability and volume fraction.

## Materials and Methods

### Datasets

All brain tissue datasets used in this study were segmented by the authors of the respective publications. The rat brain dataset was obtained from the work of Kinney et al. [27], who shared electron microscopy data from the CA1 region of the hippocampus. The mouse brain dataset was sourced from Karlupia et al. [30], who performed immersion fixation on mouse brain tissue and acquired a sample from a depth of 1 mm within the cerebral cortex. The human brain dataset was obtained from Shapson-Coe et al. [31], who shared a 1 mm^3^ sample of human brain tissue obtained from the left anterior middle temporal gyrus, a region of the cerebral cortex. All tissue samples were obtained from gray matter. The ECS pathway was extracted by performing a Boolean difference operation in COMSOL. Supplementary Table 1 summarizes basic information about the three datasets analyzed in this work. The rat’s electron microscopy dataset is publicly available [27]. The mouse and human datasets can be downloaded using the Neuroglancer links shared in the respective papers [30, 31].

### CFD simulations

In order to calculate the permeability, we first performed a pressure driven steady creeping flow simulation of interstitial fluid (ISF) through the ECS in COMSOL. We imposed a pressure gradient |∇P|=1 mmHg/mm as used by Holter et al. [26]. The use of the creeping flow approximation is justified because the Reynolds number of flow in the ECS is approximately 10^−3^ [32]. For the rat, mouse, and human simulations, we verified that the computational meshes were converged and the results were insensitive to further mesh refinement, as shown in Supplementary Fig. 1. Then we determined the Darcy velocity $\left({\vec{u}}_{D}\right)$ at the outlet and solved for the permeability *k* using Darcy’s equation:

(1) $$k=\frac{\left|{\vec{u}}_{D}\right|\mu }{\left|\nabla P\right|},$$

where μ=0.8mPa⋅s is the dynamic viscosity of ISF (assumed to match that of water). We set the fluid density to be $1000\;kg/m^{3}$. We imposed slip boundary conditions on exterior faces and no-slip boundary conditions on interior walls. We determined ${\vec{u}}_{D}$ by assuming it to be aligned with the pressure gradient and dividing the volume flow rate at the outlet by the area of the outlet. The volume flow rate at the outlet (Q) was determined by integrating the normal velocity component over the outlet surface:

(2) $$Q=\int_{A}\vec{v}\cdot \hat{n}\;dA,$$

where $\vec{v}$ is the local fluid velocity and $\overset{ˆ}{n}$ is the outward unit vector normal to the outlet surface. To assess anisotropy, we changed the direction of the imposed pressure drop. We calculated the volume fraction as the ratio of the wetted volume to the total tissue volume. All considered samples had volume fractions ranging from 0.12 to 0.30.

A representative elementary volume (REV) is the minimum volume of a porous medium over which a structural or transport property (like porosity or permeability) becomes independent of the domain size being considered. We determined the REV to be 2 μm^3^, 1 μm^3^ and 1 μm^3^ for the rat, mouse, and human datasets (Supplementary Fig. 2), respectively. We found that the permeability changes by less than 12% in rat data, 5% in mouse data, and 47% in human data for volumes equal to or larger than the REV.

### Scaling astrocyte and Uniform scaling

To expand or contract the cellular features, we scaled the individual cells about their centroid by a factor in SolidWorks. This allowed us to achieve ECS pathways with various volume fractions. This method leads to uniform variation of ECS. For astrocyte scaling, we changed the sizes of only the astrocytes and kept the size of other cellular features fixed.

### Empirical formula

To obtain an empirical formula relating the volume fraction and the permeability, the CFD derived data were fitted using a second-order polynomial regression model in MATLAB. The relationship between volume fraction (ϕ) and permeability (k) was approximated as $k=a\phi^{2}+b\phi +c$, where *a*, *b*, and *c* are coefficients obtained by least-squares fitting.

### Hydrualic tortuosity and Specific surface area measurement

The hydraulic tortuosity τ was computed as the ratio of the volume-averaged fluid velocity magnitude ⟨v⟩ to the volume-averaged velocity component in the direction of the applied pressure drop $\left\langle v_{i}\right\rangle$ [33]:

(3) $$\tau =\frac{\langle v\rangle }{\langle v_{i}\rangle }.$$

This definition is appropriate for fluid flow and permeability. In contrast, solute diffusion is commonly characterized by the diffusive tortuosity, $\lambda =\sqrt{D/D*}$, where *D* and D* denote the free and effective diffusion coefficients, respectively. Furthermore, tortuosity can be quantified using several approaches, including geometric tortuosity, which is defined as the ratio of the effective path length to the corresponding straight-line distance through the porous medium. Further details on the different approaches for measuring tortuosity can be found in [34]. The specific surface area (*S*) was calculated as the ratio of the wetted surface area to the wetted volume. Both the hydraulic tortuosity and specific surface area were calculated using COMSOL. The variation of specific surface area with volume fraction is shown in Supplementary Fig. 3.

### Extracellular matrix (ECM) units

To represent the extracellular matrix (ECM) within the ECS, we introduced 784 cylindrical ECM units, each with a radius of 5 nm and a length of 0.8 μm. The ECM units were oriented perpendicular to the imposed pressure gradient. Using the Boolean difference operation in COMSOL, the ECM units were subtracted from the ECS domain to represent the volume occupied by the ECM within the extracellular space. Simulations were then performed using the same parameters as described above. Increasing the number of ECM units further reduced the permeability.

### Mesoscale model

For the mesoscale models, simulations were performed using COMSOL Multiphysics. Each simulation consisted of two steps: solving for the fluid velocity field and then solving the advection-diffusion equation for solute concentration. Both the ECS and PVS were modeled as porous media, with distinct permeabilities assigned to each region. In all simulations, the dynamic viscosity was set to *μ* = 0.7 mPa·s . The mesoscale models were validated against analytical solutions of one-dimensional advection-diffusion problems, showing good agreement. Mesh sensitivity studies confirmed mesh-independent results, and the mesh used in final simulations had element sizes ranging from 1 to 25 μm.

## Results

### Interspecies permeability estimates

In Fig. 1A, we show the computational mesh constructed for simulating flow in the human brain tissue sample obtained from Shapson-Coe et al. [31]. A pressure gradient of 1 mmHg/mm is imposed for all of the simulations [26]. Figure 1B shows the resulting pressure distribution within and between some of the cells in the sample, and 1C shows the velocity magnitude at different *y* – *z* slices, where *y* and *z* are coordinates as indicated.

Figure 2A shows the permeability estimates across different species and anatomical locations, each calculated from a simulation like the example shown in Fig. 1. The permeability was quantified at five anatomical locations in the rat brain, at three locations in the mouse brain, and at four locations in the human brain. A pressure gradient was imposed along each of three Cartesian coordinate axes (*x*, *y*, *z*) and the corresponding permeabilities $\left(k_{x},k_{y},k_{z}\right)$ were quantified and are shown in Fig. 2A. Averaged across the sampled locations and directions, the permeability was 17.7 ± 4.7 nm^2^ for rat brain tissue, 34.2 ± 16.5 nm^2^ for mouse brain tissue, and 10.2 ± 7 nm^2^ for human brain tissue (all given as mean ± standard deviation). Human brain tissue exhibited lower permeability than rodent brain tissue. To assess whether ECS permeability was conserved among the three groups, we compared measurements from rat, mouse, and human brain tissue (Fig. 2A) using a rank-based analysis of covariance (ANCOVA) model to account for differences in volume fraction. Rank-based ANCOVA revealed a significant effect of group on permeability after accounting for volume fraction (*p* = 0.0015), indicating that differences in permeability persisted after accounting for differences in volume fraction. The differences may arise from differences in species, brain region, or fixation method.

### Variation of permeability with ECS volume fraction

Xie et al. [8] reported that the ECS volume fraction in the mouse brain increases from 14.1% during wakefulness to 23.4% during sleep, representing an approximately 66% expansion of the ECS. Deng et al. [35] reported that the ECS volume fraction in the mouse brain is larger under isoflurane anesthesia than in the awake state. In their experiments, the volume fraction expanded from 11% during wakefulness to 15% after 30 minutes of isoflurane exposure; the volume fraction returned to 11% 30 minutes after isoflurane was withdrawn. In contrast, the tortuosity remained largely stable across state transitions. Although these studies established that ECS volume fraction varies dynamically across physiological states, the corresponding changes in ECS permeability have not been systematically quantified. To address this question, we computationally scaled the ECS uniformly and quantified the resulting changes in ECS permeability through simulation. As shown in Fig. 2B–D, permeability increased monotonically with increasing ECS volume fraction across all species. Based on simulation results, increasing the ECS volume fraction from 13% to 25% increased permeability by 489% (5.9 fold) in rat brain tissue, by 1114% (12.1 fold) in mouse brain tissue, and by 797% (8.9 fold) in human brain tissue. These findings demonstrate that relatively modest changes in ECS volume fraction lead to substantial increases in ECS permeability, suggesting that physiological modulation of ECS geometry may strongly influence interstitial transport, as asserted by prior studies [8, 16, 36].

### Comaprison between CFD and Kozeny-Carman results

The Kozeny-Carman equation is widely used to relate the permeability *k* to the volume fraction ϕ, tortuosity τ, and specific surface area *S* of a porous medium [37]:

(4) $$k=\frac{\phi^{3}}{\tau S^{2}{\left(1-\phi \right)}^{2}}.$$

This equation, however, was originally developed for an idealized packed bed of spheres and assumes a homogeneous, isotropic porous medium [38]. Because brain tissue exhibits a heterogeneous cellular microstructure [39], it is important to evaluate the applicability of this idealized relationship for predicting ECS permeability. We therefore used the Kozeny–Carman equation to estimate ECS permeability and evaluated its agreement with the permeability obtained from our CFD simulations.

The hydraulic tortuosity *τ* was computed as the ratio of the volume-averaged fluid velocity magnitude to the volume-averaged velocity component in the direction of the applied pressure drop [33]. Mathematically, we can express hydraulic tortuosity by:

(5) $$\tau =\frac{\langle v\rangle }{\langle v_{i}\rangle }.$$

The specific surface area (*S*) was calculated as the ratio of the wetted surface area to the wetted volume. Figures 2B–D compare the permeability predicted by the Kozeny-Carman equation with the values obtained from the simulations for different species. The Kozeny-Carman equation consistently underestimates the permeability relative to the simulation by 62 ± 19%, 59 ± 20%, and 66 ± 25% (mean ± standard deviation) for the rat, mouse, and human datasets, respectively. This systematic underestimation indicates that the assumptions underlying the classical model do not adequately capture the complex microstructural features governing fluid transport in brain tissue.

### Empirical relationship between ECS permeability and volume fraction

To better capture the observed dependence of permeability on volume fraction, we used an empirical second-order polynomial fit (dashed black lines in Fig. 2B–D), given by

(6) $$k=a\phi^{2}+b\phi +c,$$

where *a*, *b*, and *c* are the quadratic, linear, and constant fit coefficients, respectively. The fitted coefficient values for each dataset are reported in Table 1.

Across the range of volume fractions relevant to brain tissue, the polynomial fit yielded lower in-sample errors than the Kozeny-Carman relationship. For the considered ECS volume fractions, the predictions of the empirical relation were compared with the CFD results. Using the empirical relation reduced the mean error relative to the simulations from 62% to 17%, 59% to 13%, and 66% to 26% for the rat, mouse, and human brain tissue datasets, respectively. Importantly, this empirical relationship is intended to describe permeability over the range of volume fractions relevant to brain tissue across sleep-wake states, approximately (0.13≤ϕ≤0.27).

The Kozeny-Carman equation (Eq. 4) estimates that permeability would increase by 2105% (22 fold), 3009% (31 fold), and 3856% (39.5 fold), respectively, for the same changes of volume fraction. However, if we keep the specific surface area and tortuosity constant when using the Kozeny–Carman equation, the estimated permeability would change by 9.5 fold as the volume fraction increases from 0.13 to 0.25. This indicates that much of the observed change arises from changes in the specific surface area and tortuosity. Our empirical relation estimates that when the volume fraction varies from 0.13 to 0.25, permeability increases by 7.9 fold, 20.3 fold, and 28.6 fold for the rat, mouse, and human brain tissue datasets, respectively.

### Uniform scaling versus Astrocyte scaling

Previous studies have suggested that astrocyte swelling modulates the ECS volume fraction [27]. Recently, Deng et al. [35] demonstrated that sleep-wake state-dependent fluctuations in norepinephrine levels regulate astrocyte volume, resulting in corresponding expansion or contraction of the ECS. Therefore, to explore how scaling the cells uniformly compares with scaling only the astrocytes, we also quantified the dependence of ECS permeability on ECS volume fraction when selectively varying astrocyte size. The resulting permeability–volume fraction relationship closely matched that obtained using uniform ECS scaling, indicating that both approaches yield comparable changes in permeability across the range of ECS volume fractions investigated (Supplementary Fig. 4). Therefore, our results suggest that the brain permeability could be effectively controlled by astrocytes alone, without altering neurons or other cells, suggesting that the cell types and biochemical pathways that govern brain clearance may differ from those that govern neuronal signaling.

### Mesoscale brain waste clearance model

Guo et al. [13] reported that in their advection-diffusion model of brain tissue, which included flow in the PVS and ECS, a permeability of 3000 nm^2^ could reproduce the clearance measured by Cserr et al. [29] . We refer to this computational model as the mesoscale model. This permeability is substantially higher than the mean permeability measured in rat brain tissue in our study, 17.7 nm^2^. Using the same mesoscale computational model as Guo et al. but allowing solute to be cleared not only to the subarachnoid space but also to ventricles, we were able to reproduce the clearance observed experimentally by Cserr et al. using a permeability value similar to that obtained in the present study. Figure 3A shows the updated mesoscale model, with an additional concentration-flux boundary condition applied to the bottom surface of the tissue domain. Solute is allowed to cross this bottom surface, representing clearance through the ventricular walls. Figure 3B shows the volume-averaged concentration in the ECS as it varies over time, for various values of the ECS permeability and different bottom-surface boundary conditions. The dashed black line represents the experimentally measured decay of the average concentration, normalized by its maximum value, from Cserr et al. The red and blue curves are the simulation results from Guo et al.’s mesoscale model, which did not include a concentration-flux boundary at the bottom surface. However, when the concentration-flux boundary condition is included, the simulation agrees well with the experimental data from Cserr et al. The concentration flux at the bottom surface is prescribed as $J_{c}=-k_{c}c$, which represents solute transport into an exterior domain that is not explicitly modeled and is assumed to include convective clearance. Here, *k_c_* is the mass-transfer coefficient and *c* is the solute concentration at the surface. We found that $k_{c}=1.3\times 10^{-8}m/s$, corresponding to the green curve in Fig. 3(B), provides good agreement with the experimental data.

## Discussion

Quantifying ECS permeability is a cornerstone of understanding mass transport in the brain, where both passive diffusion and advective fluid flow govern the distribution of endogenous solutes, metabolic wastes, and therapeutic agents. Methods such as convection-enhanced delivery rely on pressure gradients rather than passive diffusion alone. Hence, precise quantification of ECS permeability is vital for optimizing infusion protocols, predicting drug dispersion, and overcoming transport barriers in diseased tissue. Despite decades of investigation, reported permeability estimates span several orders of magnitude, reflecting substantial methodological differences and leaving the true permeability of the ECS unresolved. Recent advances in high spatial resolution electron microscopy, together with improved tissue fixation methods that better preserve the ECS ultrastructure, have created an unprecedented opportunity to address this longstanding uncertainty. By integrating these anatomically realistic reconstructions with simulations, our present study provides a physics-based framework for estimating ECS permeability directly from its underlying microstructure.

### Interspecies permebaility

In this work, we compared ECS permeability across three different species, and the permeability for all the species was found to be on the order of 10 nm^2^ but varied several-fold across datasets. The permeability of mouse brain tissue was roughly thrice, and that of rat brain tissue was roughly twice, that of human brain tissue. It should be noted that the rat brain tissue dataset was extracted from the hippocampus, the mouse brain tissue dataset from the cerebral cortex, and the human brain tissue dataset from the left anterior middle temporal gyrus, a region within the temporal cortex. The three different brain tissue datasets from the rat, mouse, and human brains were each acquired using different tissue chemical fixation methods that can produce different artifacts and volume fractions. However, our results suggest that permeability results are of the same order, regardless of species, brain location, or chemical fixation method, provided that the tissue volume fraction remains close to in vivo values. Future work could investigate whether the difference we observed are more attributable to differences in species, brain location, or fixation method. Perhaps brain tissue is typically less permeable in larger animals and/or in primates; future studies of other species, like non-human primates and pigs, might bring insights.

### Darcy’s velocity in the brain tissue across species

Experimentally measuring ISF velocity within the ECS remains challenging because the fluid motion is extremely slow and difficult to distinguish from diffusion within the narrow, tortuous spaces surrounding cells. Measurements are further complicated by limited spatial resolution, which can hinder discrimination between ECS flow and transport within perivascular or other fluid compartments, as well as by tracer dispersion and experimental noise. Analytical and computational models therefore provide a valuable complementary approach for estimating ISF velocity and explaining the physical mechanisms governing fluid transport within the ECS. Based on our best guess of pressure gradient (|∇*P*| = 1 mmHg/mm as used by Holter et al. [26]), averaged across the sampled locations, the Darcy or the superficial fluid velocity in the ECS was 2.7±0.9nm/s(n=5) for rat brain tissue, 6.4±3.3nm/s(n=3) for mouse brain tissue, and 1.5±0.8nm/s(n=4) for human brain tissue (all given as mean ± standard deviation for *n* independent measurements), and that value would scale linearly with the pressure gradient, which is highly uncertain. Previous studies have also reported estimates of superficial velocity of the ISF. For example, Ray et al. [11] used a finite element computational model based on porous media flow and advection–diffusion transport equations to simulate brain interstitial transport, yielding a maximum superficial velocity of approximately 800 nm/s. Holter et al. [26] estimated a superficial velocity of approximately 1 nm/s, suggesting that diffusion is the dominant mechanism of solute transport within the ECS. Using an inverse computational modeling approach, Vinje et al. [16] estimated ISF velocities in the human brain from MRI measurements to range from approximately 16.7 to 150 nm/s. Using a mesoscale computational model, Guo et al. [13] estimated an ISF velocity of approximately 3 nm/s. The MR-AIV model of Toscano et al. [17] estimated a velocity in the deep brain of approximately 100 nm/s, although the spatial resolution was 100 μm isotropic, so this estimate includes the effects of PVS flows in addition to ECS flows.

### Comparison with Kozeny-Carman equation

Numerical models [12, 40] of glymphatic flow often rely on the Kozeny-Carman equation to estimate the parenchymal permeability during different arousal states. We found that the Kozeny-Carman equation consistently underestimates ECS permeability, and modeling permeability using the Kozeny-Carman equation would tend to underestimate interstitial fluid flow rates. Moreover, when changes in tortuosity and specific surface area accompany changes in ECS volume fraction, the Kozeny–Carman relation overestimates the corresponding differences in permeability between sleep and wake states. In contrast, when volume fraction is varied without changing the specific surface area and tortuosity, the Kozeny–Carman relation predicts a sleep–wake difference in permeability comparable to the estimates from the simulations and empirical relation.

### Comparison of the present study with prior works and Mesoscale model

Holter et al. [26] reported a mean permeability of 10.7 nm^2^ for rat brain tissue using a very similar approach to the present work on a tissue chunk extracted from the same data set, which is in good agreement with the mean permeability of 17.7 nm^2^ determined in the present work. This work confirms their finding and expands on it in several important ways. First, the permeability of multiple tissue chunks was determined, providing an estimate of variation from location to location. Second, the ECS was expanded and shrunk in simulations, revealing variation of permeability with volume fraction, specific surface area, and tortuosity. And third, permeability was estimated for samples from mouse and human brains as well. Toscano et al. [17] estimated permeability across different brain regions using a machine learning model, MR-AIV. For the mouse hippocampus, they reported a permeability of 435 nm^2^ , approximately an order of magnitude higher than our estimates. This discrepancy may arise from differences in spatial scale and tissue representation, as the MR-AIV model was trained on dynamic contrast-enhanced magnetic resonance imaging data with voxel sizes of 100 μm per side. Such large voxels likely capture heterogeneous tissue compartments, including larger fluid spaces and low resistance pathways such as PVSs, rather than only representing the ECS.

Guo et al. estimated the ECS permeability to be approximately 3000 nm^2^ [13]. In that work, the authors used a mesoscale computational model to determine the permeability required to reproduce the solute clearance observed in the experiments of Cserr et al. [29]. In the Cserr et al. study, the solute was infused into the caudate nucleus, which is located immediately adjacent to the right lateral ventricle [19]. The proximity of the infusion site to the ventricle likely allowed substantial solute clearance via the ventricle because the ependymal lining separating the caudate nucleus from the ventricular space lacks the tight junctions that characterize the BBB, allowing relatively unrestricted exchange between the brain parenchyma and cerebrospinal fluid. A study by Rosenberg et al. [41] supports this hypothesis. They found that permeability of the ependymal cell layer lining the ventricle of a cat’s brain was 2.3 ± 0.4 × 10^−6^ m/s. The mesoscale computational model of Guo et al. did not explicitly account for solute exchange with the ventricular system. Consequently, a higher ECS permeability was required in their simulations to reproduce the clearance observed by Cserr et al. In the present study, we revisited the same computational model but incorporated a concentration-dependent flux boundary condition at the ventricular interface to model the transport across the ependymal lining separating the right lateral ventricle and the caudate nucleus. Using this approach, we found that a mass transfer coefficient of *k_c_* = 1.3 × 10^−8^ m/s yielded a clearance prediction that agreed well with the experimental data without requiring such a high ECS permeability. The permeability reported by Rosenberg et al. [41] was measured using tritiated water (molecular weight ≈ 20 Da), whereas Cserr et al. quantified the clearance of serum albumin (69 kDa). This difference of more than three orders of magnitude in molecular weight leads to substantially different diffusion coefficients, providing a plausible explanation for the discrepancy between the value Rosenberg et al. estimated and the one used in the present work. A sensitivity analysis was performed to quantify the effect of the mass transfer coefficient *k_c_* on predicted clearance, with the results shown in Supplementary Fig. 5. The predicted clearance was highly sensitive to *k_c_*. Small values (10^−10^ – 10^−9^ m/s) resulted in insufficient waste removal, whereas large values (10^−7^ – 10^−6^ m/s) produced clearance substantially faster than that observed experimentally.

### Variation in ECS tortuosity and permeability with volume fraction

Previous studies of the tortuosity of ECS pathways have focused primarily on measuring the diffusive tortuosity, commonly defined as $\lambda =\sqrt{D/D*}$, where *D* and D* represent the free and effective diffusion coefficients, respectively. Thorne and Nicholson [42] measured ECS diffusive tortuosity for TMA, TR-dex3, TR-dex70, and QD665, reporting values of approximately 1.6, 2.03, 2.68, and 10.78, respectively. Kinney et al. [27] reported ECS diffusive tortuosity values ranging from 1.22 to 1.42 across different ECS reconstructions at the same volume fraction. In this study, we calculated hydraulic tortuosity, defined as the ratio of the volume-averaged velocity magnitude to the volume-averaged velocity component in the direction of the imposed pressure gradient. We found hydraulic tortuosity values ranging from 1.23 to 1.34 (*n* = 4) for rat, 1.16 to 1.38 (*n* = 3) for mouse, and 1.23 to 1.36 (*n* = 4) for human brain tissue datasets, where *n* represents the number of independent measurements. Hydraulic tortuosity decreased with increasing ECS volume fraction (Supplementary Fig. 6). The hydraulic tortuosity values obtained in this study are comparable to the diffusive tortuosity values reported by Kinney et al. [27], suggesting consistency between hydraulic and diffusive measures of ECS tortuosity. However, the diffusive tortuosity of the ECS is not expected to exceed 1.22 [19]. The experimentally measured diffusive tortuosity of approximately 1.6 [42] has been attributed to additional contributions from dead spaces in the ECS, interactions with extracellular matrix components, and ISF viscosity [19].

To our knowledge, no previous study has used electron microscopy data to quantify how ECS permeability varies with the changes in volume fraction that occur between the awake and sleep states. Experiments by Xie et al. [8] demonstrated that the increase in ECS volume fraction during sleep, compared with the awake state, correlates with enhanced interstitial fluid flow and efficient waste clearance from the brain. Consistent with these findings, our results predict an approximately 500% increase (or more) in permeability as the ECS volume fraction increases from approximately 13% in the awake state to about 25% during sleep across all three species. This substantial increase in permeability suggests that interstitial fluid can flow more readily through the ECS during sleep, providing a mechanistic explanation for the enhanced waste clearance observed experimentally.

### Gray matter and white matter permeability

Brain tissue can be broadly classified into gray matter and white matter based on cellular composition and microstructure. The three-dimensional brain tissue datasets analyzed in this study were obtained exclusively from gray matter regions. Because gray matter has a higher cellular density and, consequently, a lower extracellular volume fraction than white matter, it is expected to exhibit lower permeability. Averaged across the sampled locations, the mean permeability of rat brain tissue along the three orthogonal Cartesian axes was 20.6, 19.3, and 17.5 nm^2^, respectively. For mouse brain tissue, the corresponding mean permeabilities were 26.4, 44.9, and 31.3 nm^2^, whereas those for human brain tissue were 8.9, 9.6, and 12.1 nm^2^, respectively (see Supplementary Fig. 7). Simulations by Yuan et al. [43] showed that the permeability of the corona radiata in sheep white matter was 3129 nm^2^ parallel to the axons and 27 nm^2^ perpendicular to the axons, indicating strong anisotropy. Previous studies have debated whether the relatively low permeability of the ECS is sufficient to support advective transport, with several arguing against a significant role for advection in brain waste clearance. Our findings suggest that this debate should consider regional heterogeneity in brain tissue. Because ECS permeability varies substantially between gray and white matter, the relative contributions of advection and diffusion to solute transport are also likely to differ across brain regions. Further, PVSs form low-resistance fluid pathways that facilitate CSF flow through the brain parenchyma, leading to the clearance of interstitial solutes, including amyloid-*β* [5]. Consequently, waste clearance throughout the entire brain is unlikely attributable to a single transport mechanism: advection and diffusion are likely both important.

### Limitations and Future works

Our computational approach for modifying the ECS volume fraction has several limitations. First, uniform scaling of the cellular geometry does not fully capture the structural remodeling that occurs in vivo during physiological changes in ECS volume fraction. In Supplementary Fig. 4, the comparison between astrocyte-specific and uniform scaling is based on a domain containing only a fragment of an astrocyte, as simulating an entire astrocyte would be computationally expensive. Thus, the results describe the local effects of astrocytic size variation rather than providing a direct comparison between uniform cellular scaling and scaling of an entire astrocyte. Second, brain tissue can undergo large nonlinear deformations and is commonly treated as a poroviscoelastic material. In all of our simulations, brain cells were assumed to be rigid solids. In a simulation, we modeled the cells as linear elastic with a Young’s modulus of 15 Pa, and this assumption increased the predicted permeability by only 1.1%, with the maximum cell surface deformation of 2.38 nm. Nevertheless, cellular deformation and fluid exchange associated with poroelastic behavior may alter interstitial flow within the ECS and thereby affect estimates of ECS permeability. Incorporating poroviscoelastic tissue mechanics into future computational models may therefore provide more physiologically realistic predictions of transport through the brain ECS.

Our study used the segmented datasets provided by the authors of the corresponding studies [27, 30, 31]. The fidelity with which the ECS is preserved depends strongly on the tissue fixation protocol and on whether osmotic agents are used during sample preparation. Consequently, artifacts related to fixation and segmentation may influence the estimated ECS permeability. The raw three-dimensional reconstructions acquired by Kinney et al. [27] had an ECS volume fraction of approximately 8%, substantially lower than the in vivo estimate of approximately 20%. To obtain a physiologically realistic microstructure, the authors computationally expanded the ECS to achieve a volume fraction of 20%. This observation suggests that perfusion fixation alone did not adequately preserve the native ECS. Karlupia et al. [30] used immersion fixation together with mannitol as an osmotic agent to better preserve the ECS in mouse brain tissue, thereby reducing fixation-induced artifacts. However, the dataset was generated using automated cell segmentation, which may have omitted fine cellular processes. Such omissions would be interpreted as extracellular space, potentially leading to a slight overestimation of the ECS volume fraction and, consequently, permeability. Karlupia et al. had reported an average ECS volume fraction of 15% based on a manual segmentation in the two-dimensional cross-sectional electron micrographs. In the present study, we reported the ECS volume fraction by comparing the automatically segmented ECS volume in the three-dimensional tissue volume. Due to segmentation errors, the ECS volume fractions in the analyzed chunks were greater than 15%. In contrast, Shapson-Coe et al. [31] used immersion fixation without osmotic agent, making the human brain dataset more susceptible to fixation-induced tissue shrinkage and incomplete preservation of the ECS than the data from Karlupia et al., but it still preserved the tissue better than the approach used by Kinney et al., since the volume fractions are about 19% in the tissue chunks we analyzed. Although the cellular segmentation was automatically generated and subsequently proofread, residual merge and split errors may still be present as the authors indicated. These segmentation errors could introduce artificial obstructions or spurious connections in the computational domain, thereby influencing the estimated permeability. Another potential limitation of this study is the size of the representative elementary volume, which may introduce substantial uncertainty in the estimated quantities, particularly for the human datasets (Supplementary Fig. 2). Simulations using larger tissue volumes could help reduce this uncertainty and provide more robust estimates.

Our results also do not account for the role of extracellular matrix (ECM) components in modulating ECS permeability. For a first-order estimate, we performed simulations in which the ECM was represented by cylindrical structures with a radius of 5 nm (Supplementary Fig. 8). These structures were introduced into a model of rat brain tissue having ECS volume fraction of 0.26, a hydraulic tortuosity of 1.21, and a permeability of 23.93 nm^2^ . To maintain the same ECS volume fraction before and after ECM incorporation, we rescaled the ECS accordingly. Incorporation of the ECM reduced the permeability by approximately 6.5 fold, whereas the hydraulic tortuosity remained largely unchanged. However, further experimental characterization of the ECM is needed to more accurately model and understand its effects on tortuosity and permeability.

Future work could estimate ECS permeability using tissue samples that better preserve the native extracellular space, which would improve the accuracy of permeability estimates. Incorporating these permeability measurements into computational models of interstitial fluid flow and solute transport will provide a better understanding of the relative roles of advection and diffusion in brain waste clearance. We varied the direction of the imposed pressure drop in order to detect a possible directional dependence of permeability (results shown in Supplementary Fig. 7). Our results indicate that a larger sample size is needed to conclude whether the brain tissue is isotropic, but at least the measurements are within an order of magnitude of each other, unlike white matter [44, 45].

## Conclusions

In conclusion, our study provides a quantitative characterization of the ECS permeability of brain gray matter, based on realistic three-dimensional electron microscopy reconstructions. We found that ECS permeability differed significantly between different data sets. However, the magnitude of these differences was within a few-fold and did not span an order of magnitude. Permeability increases with ECS volume fraction, enabling estimation of permeability under different arousal states, including wakefulness and sleep. We further demonstrate that the classical Kozeny-Carman equation substantially underestimates brain ECS permeability, and we propose a simple empirical relationship that more accurately captures the dependence of brain tissue permeability on extracellular space volume fraction. Moreover, similar permeability trends obtained from uniform and astrocyte scaling suggest that the mechanism of volume fraction change has little effect on bulk permeability. Finally, by incorporating the predicted permeability into a computational model of interstitial transport, we demonstrate its utility for simulating solute clearance in brain tissue, providing a framework for multiscale models of fluid flow and waste transport in the central nervous system.

## Supplementary Material

This is a list of supplementary files associated with this preprint. Click to download.

- SIFBCNSECSPermeability.pdf

Supplementary figures have been included with this file.

## Figures and Tables

**Fig. 1:**
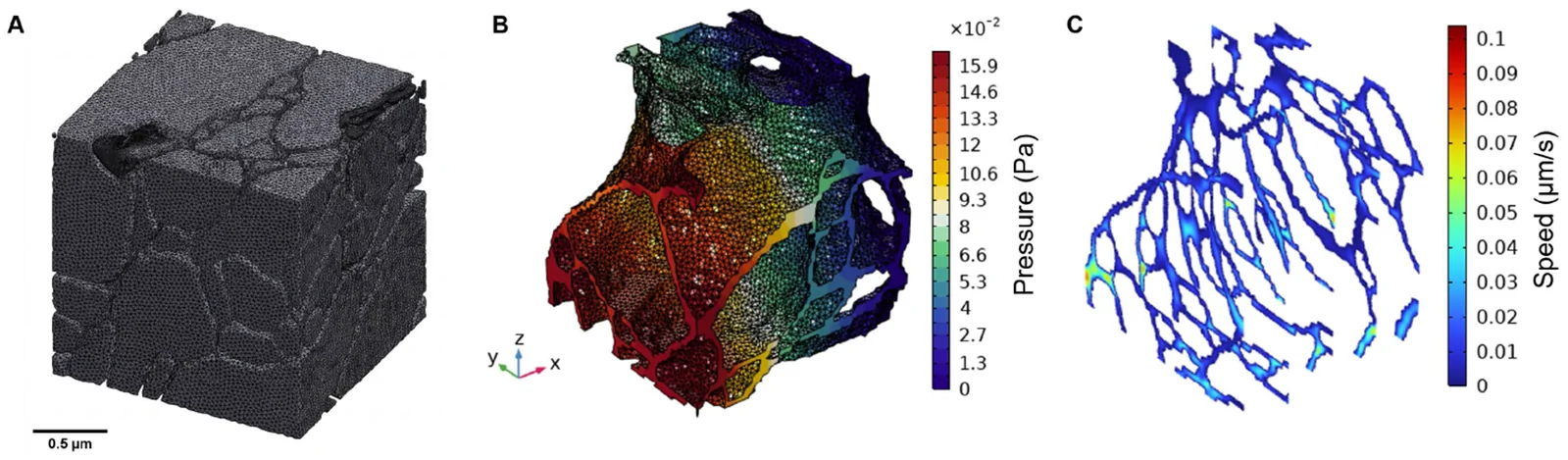
**A**, Reconstruction of a sample of human brain tissue (data from Shapson-Coe et al. [31]). **B**, Simulated pressure distribution within the extracellular space (ECS) pathway, along with the computational mesh. **C**, Speed on different cross sections of the ECS.

**Fig. 2:**
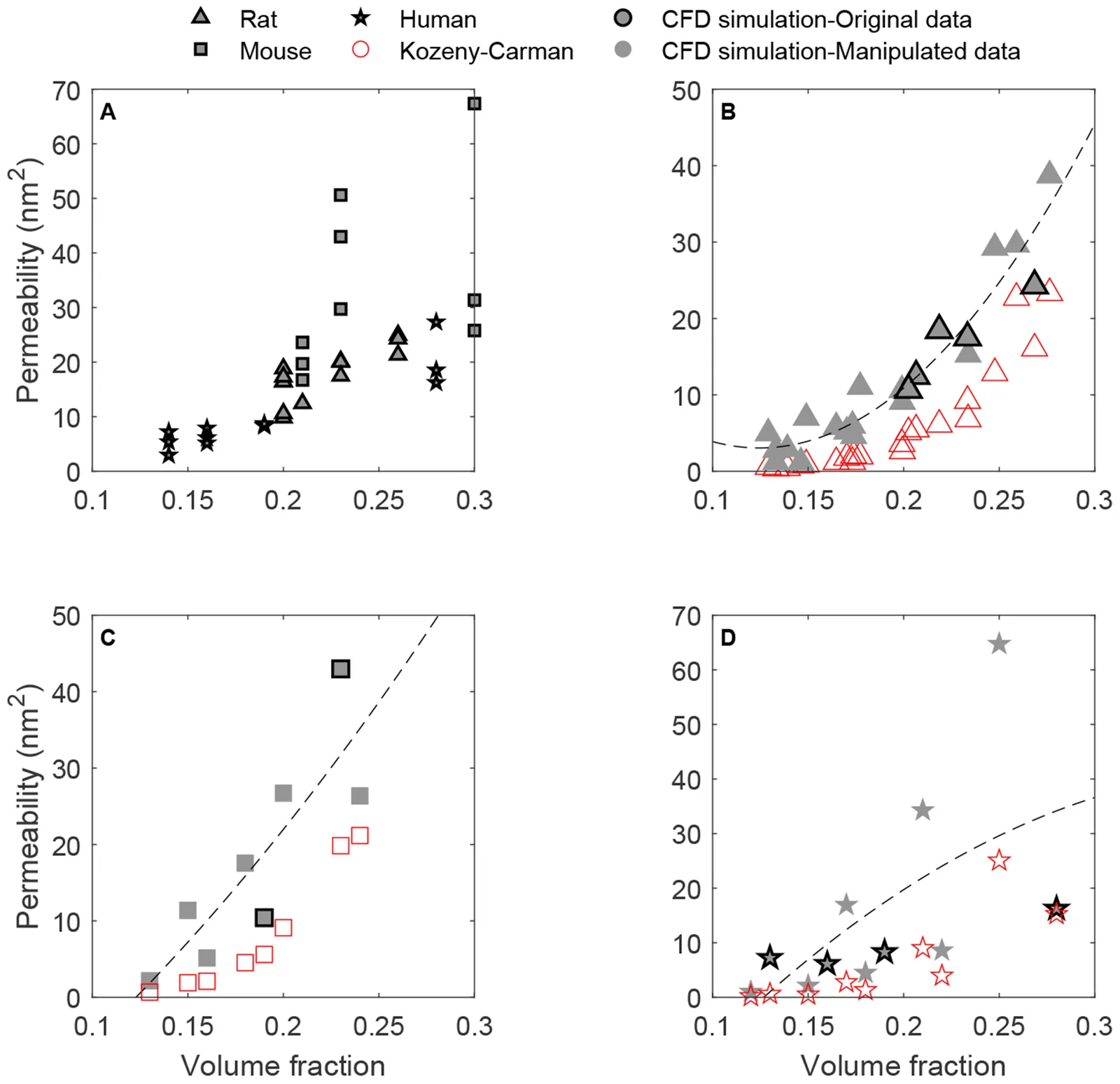
**A**, Permeability of brain tissue samples from different species and different directions of imposed pressure gradient. **B–D**, Comparison between simulation and Kozeny-Carman results for rat [27] (B), mouse [30] (C), and human [31] (D) brain tissue. Markers with black edges indicate results from original extracellular space (ECS) data. Red markers indicate results from the Kozeny-Carman equation. Markers without edges indicate results in which ECSs were computationally scaled. The dashed black line represents a second-order polynomial fit to the permeability estimates obtained from the simulations. Our results indicate that ECS permeability is of the same order of magnitude across datasets sampled from three distinct species. The Kozeny–Carman equation consistently underestimated ECS permeability, while the empirical relation better predicted the permeability.

**Fig. 3:**
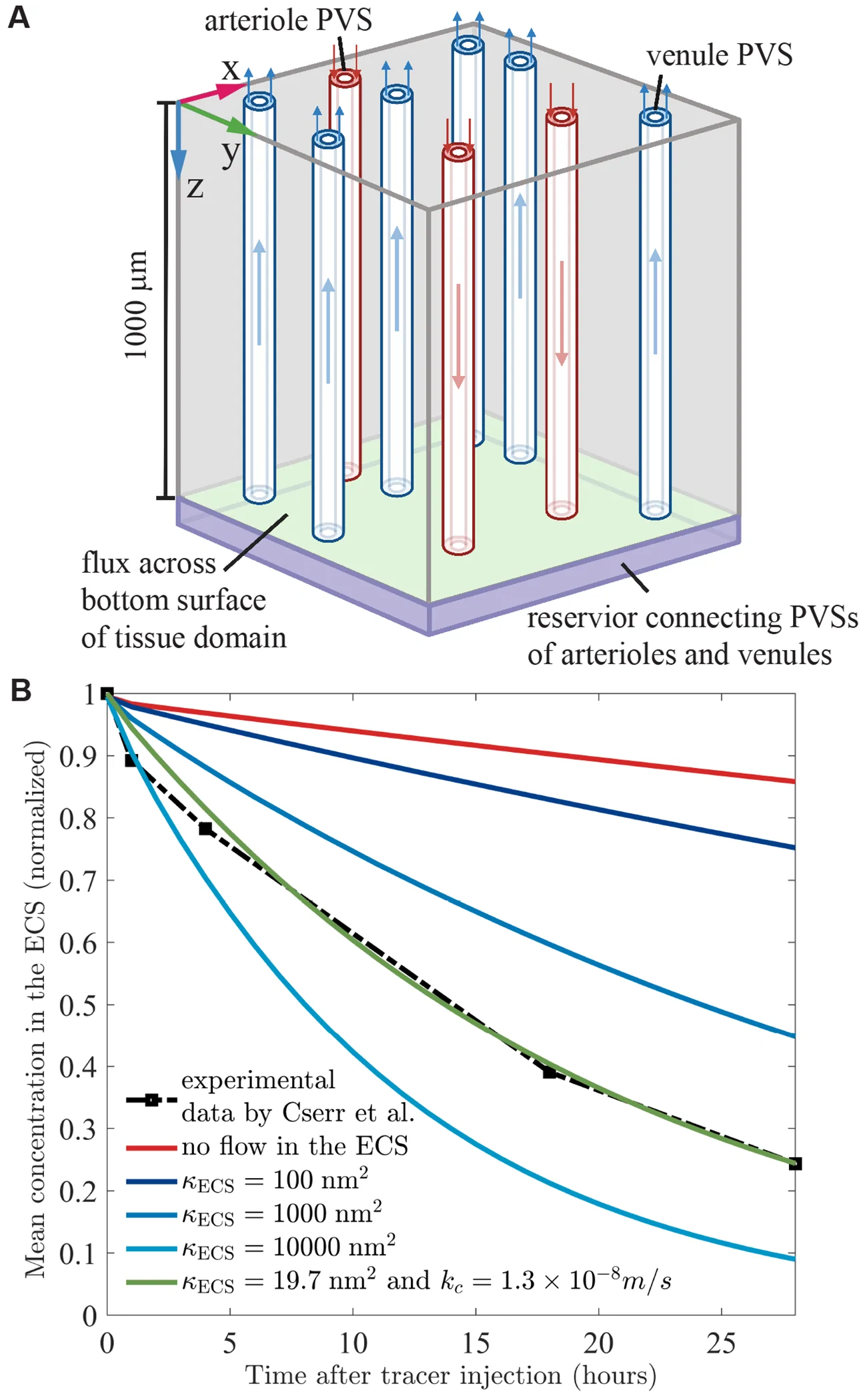
**A**, Schematic of mesoscale model: the advection-diffusion model of brain tissue that included flow in the perivascular space (PVS) and extracellular space (ECS). Cerebrospinal fluid (CSF) flows through an array of PVSs of arterioles (red) and venules (blue), connected by a reservoir of small volume (purple). The arrows show the flow direction, and fluid also flows through the ECS, entering from arteriole PVSs and exiting to venule PVSs. A concentration-flux boundary condition is applied to the bottom surface of the tissue domain (green). **B**, Time evolution of the volume-averaged solute concentration in the ECS.

**Table 1:** Coefficients of the empirical relationship.

Species	a (nm^2^)	b (nm^2^)	c (nm^2^)	*R* ^2^
Rat	1376.0	−342.8	24.4	0.92
Mouse	380.3	162.1	−25.6	0.73
Human	−579.6	458.7	−48.8	0.37

## Data Availability

The datasets supporting the conclusions of this article are included within the article and its Supplementary Information.

## Materials availability

Code availability

The code used to download the brain tissue dataset and convert it into a surface mesh file, along with a sample brain tissue ‘.stl’ file for each species, is available at https://github.com/aditya22-FluidLab/Brain-tissue.

## References

[1] Damkier H. H., Brown P. D. & Praetorius J. Cerebrospinal fluid secretion by the choroid plexus. Physiological reviews (2013). 10.1152/physrev.00004.2013 24137023

[2] Rasmussen M. K., Mestre H. & Nedergaard M. Fluid transport in the brain. Physiol. Rev. (2021). 10.1152/physrev.00031.2020 PMC8897154 33949874

[3] Gan Y. Perivascular pumping of cerebrospinal fluid in the brain with a valve mechanism. Journal of the Royal Society Interface 20, 20230288 (2023). 10.1098/rsif.2023.0288 PMC10509587 37727070

[4] Causemann M., Enger R. & Rognes M. E. Stretch and flow at the gliovascular interface: High-fidelity modeling of astrocyte endfeet. Proceedings of the National Academy of Sciences 123, e2517059123 (2026). 10.1073/pnas.2517059123 PMC12994201 41824500

[5] Iliff J. J. A paravascular pathway facilitates csf flow through the brain parenchyma and the clearance of interstitial solutes, including amyloid *β*. Science Translational Medicine 4 (2012). URL https://pubmed.ncbi.nlm.nih.gov/22896675/. 10.1126/scitranslmed.3003748 PMC3551275 22896675

[6] Mestre H. Flow of cerebrospinal fluid is driven by arterial pulsations and is reduced in hypertension. Nature communications 9, 4878 (2018). 10.1038/s41467-018-07318-3 PMC6242982 30451853

[7] van Veluw S. J. Vasomotion as a driving force for paravascular clearance in the awake mouse brain. Neuron 105, 549–561 (2020). 10.1016/j.neuron.2019.10.033 PMC7028316 31810839

[8] Xie L. Sleep drives metabolite clearance from the adult brain. Science 342, 373–377 (2013). 10.1126/science.1241224 PMC3880190 24136970

[9] Hickman S., Izzy S., Sen P., Morsett L. & El Khoury J. Microglia in neurodegeneration. Nature neuroscience 21, 1359–1369 (2018). 10.1038/s41593-018-0242-x PMC6817969 30258234

[10] Tarasoff-Conway J. M. Clearance systems in the brain—implications for alzheimer disease. Nature reviews neurology 11, 457–470 (2015). 10.1038/nrneurol.2015.119 PMC4694579 26195256

[11] Ray L., Iliff J. J. & Heys J. J. Analysis of convective and diffusive transport in the brain interstitium. Fluids Barriers CNS 16, 6 (2019). 10.1186/s12987-019-0126-9 PMC6402182 30836968

[12] Tithof J. A network model of glymphatic flow under different experimentally-motivated parametric scenarios. iScience 25, 104258 (2022). URL https://www.sciencedirect.com/science/article/pii/S2589004222005284. 10.1016/j.isci.2022.104258 PMC9062681 35521514

[13] Guo Y., Quirk K., Kelley D. H. & Thomas J. H. Advection and diffusion in perivascular and extracellular spaces in the brain. Journal of The Royal Society Interface 22, 20250010 (2025). 10.1098/rsif.2025.0010 PMC12092104 40393523

[14] Kedarasetti R. T., Drew P. J. & Costanzo F. Arterial vasodilation drives convective fluid flow in the brain: a poroelastic model. Fluids and Barriers of the CNS 19, 34 (2022). 10.1186/s12987-022-00326-y PMC9107702 35570287

[15] Bork P. A. Modeling of brain efflux: constraints of brain surfaces. Proceedings of the National Academy of Sciences 121, e2318444121 (2024). 10.1073/pnas.2318444121 PMC11032467 38598340

[16] Vinje V. Human brain solute transport quantified by glymphatic mri- informed biophysics during sleep and sleep deprivation. Fluids and Barriers of the CNS 20, 62 (2023). 10.1186/s12987-023-00459-8 PMC10439559 37596635

[17] Toscano J. D. Mr-aiv reveals in vivo brain-wide fluid flow with physics-informed ai. Science Advances 12, eaeb0404 (2026). 10.1126/sciadv.aeb0404 PMC13215179 42202031

[18] Vaezi M. Robust mr-aiv: A systematic study of robustness improvement and sensitivity analysis of mr-aiv. bioRxiv (2026).

[19] Syková E. & Nicholson C. Diffusion in brain extracellular space. Physiological Reviews 88, 1277–1340 (2008). URL 10.1152/physrev.00027.2007. PMC2785730 18923183

[20] Sykova E. Extrasynaptic volume transmission and diffusion parameters of the extracellular space. Neuroscience 129, 861–876 (2004). 10.1016/j.neuroscience.2004.06.077 15561404

[21] Basser P. J. Interstitial pressure, volume, and flow during infusion into brain tissue. Microvasc. Res. 44, 143–165 (1992). 10.1016/0026-2862(92)90077-3 1474925

[22] Nagashima T., Tamaki N., Matsumoto S., Horwitz B. & Seguchi Y Biomechanics of hydrocephalus: a new theoretical model. Neurosurgery 21, 898–904 (1987). 10.1227/00006123-198712000-00019 3437958

[23] Morrison P. F., Chen M. Y., Chadwick R. S., Lonser R. R. & Oldfield E. H. Focal delivery during direct infusion to brain: role of flow rate, catheter diameter, and tissue mechanics. American Journal of Physiology-Regulatory, Integrative and Comparative Physiology 277, R1218–R1229 (1999). 10.1152/ajpregu.1999.277.4.R1218 10516265

[24] Chen M. Y., Lonser R. R., Morrison P. F., Governale L. S. & Oldfield E. H. Variables affecting convection-enhanced delivery to the striatum: a systematic examination of rate of infusion, cannula size, infusate concentration, and tissue—cannula sealing time. Journal of neurosurgery 90, 315–320 (1999). 10.3171/jns.1999.90.2.0315 9950503

[25] Jin B.-J., Smith A. J. & Verkman A. S. Spatial model of convective solute transport in brain extracellular space does not support a “glymphatic” mechanism. Journal of General Physiology 148, 489–501 (2016). 10.1085/jgp.201611684 PMC5129742 27836940

[26] Holter K. E. Interstitial solute transport in 3D reconstructed neuropil occurs by diffusion rather than bulk flow. Proc. Nat. Acad. Sci. 114, 9894–9899 (2017). 10.1073/pnas.1706942114 PMC5604020 28847942

[27] Kinney J. P. Extracellular sheets and tunnels modulate glutamate diffusion in hippocampal neuropil. Journal of Comparative Neurology 521, 448–464 (2013). URL https://onlinelibrary.wiley.com/doi/abs/10.1002/cne.23181. 10.1002/cne.23181 PMC3540825 22740128

[28] Jamal A. Infusion mechanisms in brain white matter and their dependence on microstructure: An experimental study of hydraulic permeability. IEEE Transactions on Biomedical Engineering 68, 1229–1237 (2020). 10.1109/TBME.2020.3024117 32931425

[29] Cserr H. F., Cooper D. N., Suri P. K. & Patlak C. S. Efflux of radiolabeled polyethylene glycols and albumin from rat brain. Am. J. Physiol.–Renal 240, F319–F328 (1981). 10.1152/ajprenal.1981.240.4.F319 7223889

[30] Karlupia N. Immersion fixation and staining of multicubic millimeter volumes for electron microscopy–based connectomics of human brain biopsies. Biological Psychiatry 94, 352–360 (2023). 10.1016/j.biopsych.2023.01.025 PMC10397365 36740206

[31] Shapson-Coe A. A petavoxel fragment of human cerebral cortex reconstructed at nanoscale resolution. Science 384, eadk4858 (2024). 10.1126/science.adk4858 PMC11718559 38723085

[32] Kelley D. H. & Thomas J. H. Cerebrospinal fluid flow. Annual Review of Fluid Mechanics 55, 237–264 (2023). URL 10.1146/annurev-fluid-120720-011638. PMC11651633 39691763

[33] Duda A., Koza Z. & Matyka M. Hydraulic tortuosity in arbitrary porous media flow. Phys. Rev. E 84, 036319 (2011). URL https://link.aps.org/doi/10.1103/PhysRevE.84.036319. 10.1103/PhysRevE.84.036319 22060504

[34] Holzer L. Tortuosity and Microstructure Effects in Porous Media: Classical Theories, Empirical Data and Modern Methods Vol. 333 of Springer Series in Materials Science (Springer, Cham, Switzerland, 2023). URL 10.1007/978-3-031-30477-4.

[35] Deng S. Astrocyte cell volume dynamics across cortical states and transitions. Neuron (2026). 10.1016/j.neuron.2026.03.029 42013853

[36] Kroesbergen E. Glymphatic clearance is enhanced during sleep. bioRxiv 2024–08 (2024).

[37] Thomas J. H. Theoretical analysis of wake/sleep changes in brain solute transport suggests a flow of interstitial fluid. Fluids and Barriers of the CNS 19, 30 (2022). URL 10.1186/s12987-022-00325-z. PMC9009049 35418142

[38] Carman P. C. Fluid flow through granular beds. Trans. Inst. Chem. Eng. 15, 150–166 (1937).

[39] Gevertz J. L. & Torquato S. A novel three-phase model of brain tissue microstructure. PLoS computational biology 4, e1000152 (2008). 10.1371/journal.pcbi.1000152 PMC2495040 18704170

[40] Quirk K., Boster K. A., Tithof J. & Kelley D. H. A brain-wide solute transport model of the glymphatic system. Journal of the Royal Society Interface 21, 20240369 (2024). 10.1098/rsif.2024.0369 PMC11496954 39439312

[41] Rosenberg G. A., Kyner W. T., Fenstermacher J. D. & Patlak C. S. Effect of vasopressin on ependymal and capillary permeability to tritiated water in cat. American Journal of Physiology-Renal Physiology 251, F485–F489 (1986). 10.1152/ajprenal.1986.251.3.F485 3752258

[42] Thorne R. G. & Nicholson C. In vivo diffusion analysis with quantum dots and dextrans predicts the width of brain extracellular space. Proceedings of the National Academy of Sciences 103, 5567–5572 (2006). 10.1073/pnas.0509425103 PMC1459394 16567637

[43] Yuan T., Zhan W. & Dini D. Linking fluid-axons interactions to the macroscopic fluid transport properties of the brain. Acta Biomaterialia 160, 152–163 (2023). 10.1016/j.actbio.2023.02.010 36781040

[44] Vidotto M., Bernardini A., Trovatelli M., De Momi E. & Dini D. On the microstructural origin of brain white matter hydraulic permeability. Proceedings of the National Academy of Sciences 118, e2105328118 (2021). 10.1073/pnas.2105328118 PMC8433514 34480003

[45] Bernardini A. Imaging and reconstruction of the cytoarchitecture of axonal fibres: Enabling biomedical engineering studies involving brain microstructure. Frontiers (2021).

